# The effects of improving hospital physicians working conditions on patient care: a prospective, controlled intervention study

**DOI:** 10.1186/1472-6963-13-401

**Published:** 2013-10-09

**Authors:** Matthias Weigl, Severin Hornung, Peter Angerer, Johannes Siegrist, Jürgen Glaser

**Affiliations:** 1 Institute and Outpatient Clinic for Occupational, Social, and Environmental Medicine, Ludwig-Maximilians-University Munich, Ziemssenstrasse 1, D-80336 Munich, Germany; 2 Institute of Psychology, University of Innsbruck, Innsbruck, Austria; 3 Institute for Occupational Medicine and Social Medicine, Medical Faculty, University of Düsseldorf, Düsseldorf, Germany; 4 Institute of Medical Sociology, Medical Faculty, University of Düsseldorf, Düsseldorf, Germany

**Keywords:** Hospital, Physicians, Work conditions, Intervention, Trial control study, Patient care

## Abstract

**Background:**

Physicians, particularly in hospitals, suffer from adverse working conditions. There is a close link between physicians’ psychosocial work environment and the quality of the work they deliver. Our study aimed to explore whether a participatory work-design intervention involving hospital physicians is effective in improving working conditions and quality of patient care.

**Methods:**

A prospective, controlled intervention study was conducted in two surgical and two internal departments. Participants were 57 hospital physicians and 1581 inpatients. The intervention was a structured, participatory intervention based on continuous group meetings. Physicians actively analyzed problematic working conditions, developed solutions, and initiated their implementation. Physicians’ working conditions and patients’ perceived quality of care were outcome criteria. These variables were assessed by standardized questionnaires. Additional data on implementation status were gathered through interviews.

**Results:**

Over the course of ten months, several work-related problems were identified, categorized, and ten solutions were implemented. Post-intervention, physicians in the intervention departments reported substantially less conflicting demands and enhanced quality of cooperation with patients’ relatives, compared to control group physicians. Moreover, positive changes in enhanced colleague support could be attributed to the intervention. Regarding patient reports of care quality of care, patient ratings of physicians organization of care improved for physicians in the intervention group. Five interviews with involved physicians confirm the plausibility of obtained results, provide information on implementation status and sustainability of the solutions, and highlight process-related factors for re-design interventions to improve hospital physicians work.

**Conclusions:**

This study demonstrates that participatory work design for hospital physicians is a promising intervention for improving working conditions and promoting patient quality of care.

## Background

Hospital physicians working conditions not only affect their own well-being, but to a large degree also the quality of care their patients receive [1-4]. Work overload, workflow interruptions, time pressure, conflicting demands, limited control of work, lack of participation, problems with cooperation between various professions involved in patient care, poor leadership, and low social support have been identified as critical work conditions [2,4,5]. Work stressors negatively impact work satisfaction, can lead to early career disruptions, and pose threats to physicians well-being [1,2,4-7]. Stressors impede performance in several ways, ranging from poor communication to medication errors and increased patient mortality [1,8,9].

There exists, therefore, a need for strategies to improve physicians work life. In contrast to numerous studies describing physicians job stressors, there is a void of prospective interventions aimed at promoting hospital physicians working conditions [1,10,11]. However, working conditions are responsive to improvements, especially by changing the work organization [3,12]. Organization re-design for health care workers (other than physicians) is suggested as a promising way to reduce job stressors, enhance job control, and promote patient safety [13-15].

Organization-level interventions are most likely to be successful if they follow a structured and participatory process [15-17]. This means that the physician participation is a core component of the intervention process [16]. A participatory approach makes use of local expertise, ensuring the intervention is appropriate for the specific context and enhancing physician control [14,18-20].

The present study reports a hospital-based organizational intervention involving physician participation. Specifically, we set out to conduct a prospective controlled study assessing the effects of an intervention program on physicians work conditions (as primary outcome) and quality of patient care (as secondary outcome).

## Methods

### Setting

A prospective, controlled intervention was conducted in a municipal 300-bed teaching hospital. This setting is typical for a German hospital, with regard to bed capacity, admission numbers, and number of staff, according to official statistics [21]. Four large hospital departments were included: Two internal medicine and two surgery, all of which were comparable in major organizational characteristics. Internal medicine and surgery represent conservative and operating specialties, and are by far the most frequent specialties across hospitals. The study was approved by the Ethics Committee of the Faculty of Medicine, Munich University (Nr. 124-07).

### Design and study sample

The study was designed as a prospective, controlled intervention. Prior to the intervention, the two surgical and the two internal departments were randomly assigned to an intervention and a control group, respectively. Trauma surgery and cardiology formed the intervention departments (ID), general surgery and gastroenterology the control departments (CD).

Physicians working conditions (primary outcome) and patients reported quality of care (secondary outcome) were assessed through paper-and-pencil questionnaires, both prior to baseline (T1) and after the intervention (follow-up, T2, 13 months later) identically. All physicians working in the four departments were eligible to participate in the survey. At baseline, 33 physicians worked in the ID (12 trauma, 21 cardiology) and 24 worked in the CD (11 surgery, 13 gastro-intestinal).

Patient questionnaires were filled in either during or after the hospital stay. All patients undergoing inpatient treatment and overnight stay within CD or ID were eligible. All physicians and inpatients were provided with an oral brief.

### Intervention: collective participatory work design in hospital physicians work

In the two IDs the intervention started with continuous small group meetings. Our participatory intervention adhered to the principles of the health circles or quality circles approaches, which have been shown to be effective in improving physical and psychosocial work conditions, employee well-being, and productivity [22,23]. Following the problem solving cycle, physicians voluntarily prioritized problematic working conditions, developed adequate solutions, and initiated their implementation in collaboration with colleagues. Initially, physicians were informed of the baseline assessments. Afterwards they collectively discussed problems and established a list of problematic working conditions and needs for improvement. Within each meeting, a protocol was taken to record solutions, deadlines, and responsibilities. Physicians were fully responsible for the pace of implementation. Physicians in the CD were only offered feedback regarding the baseline assessment, including information on the allocation of groups and the process of the intervention.

### Measures

#### Hospital physician working conditions

Four scales of an established, valid, and reliable German questionnaire for physicians work were applied [24]: Scale (1) Workflow interruptions are a prevalent stressor in hospital physicians’ work [25-27]; 3 items, Cronbach’s alpha coefficients (CA) .86 (T1) and .85 (T2) respectively; (2) Conflicts in role and ambiguous task demands (an important issue in physicians work [28]); 4 items, CA .77 (T1) and .81 (T2); (3) Colleague Support; 3 items, CA .77 (T1) and .79 (T2); (4) Quality losses (erroneous work or work of poor quality caused by inferior work conditions); 3 items, CA .84 (T1) and .81 (T2). Indicators 1, 2 and 4 reflect relevant work stressors (higher values mean increased risk for psychosocial stress) whereas indicator 3, colleague support, represents a valued work resource (a higher score indicates increased opportunities to deal with stress at work and/or to buffer psychological strain) [2,17,29]. Additionally, physicians were requested to evaluate the quality of cooperation with relatives of patients (1 item) and with the nursing staff (1 item). All items were answered on a five point Likert-scale.

#### Patient care quality

Patients rated the perceived quality of care using an established, standardized questionnaire (MPI) [30]. Two major aspects of quality of care were covered: (1) ‘organization of physicians care’ (4 items; e.g. “The physicians have enough time for me”); (2) ‘quality of physicians’ information’ (5 items; e.g. “Physicians provide me with detailed information regarding my medical treatment”). The 5-point Likert-scale ranged from 1 = “not at all” to 5 = “yes, very much”. CA were .74 for (1) and .79 (2) at T1, .74 (1) and .72 (2) at T2, indicating consistently acceptable reliability.

#### Process and implementation information

As a third data source, semi-structured interviews were conducted with involved physicians to obtain information on implementation and process-related factors [16,17,20]. Questions covered information such as the progress at follow-up. Three interviews with involved physicians and two with departments’ head physicians were conducted. To reduce and categorize the information, we used qualitative data analysis, i.e., to elicit main themes and summary statements [31].

### Statistical analysis

Data were analysed with PASW Statistics 19.0. Differences in subject characteristics were tested with t-tests for continuous data and Chi-Square-tests for nominal data.

To test for intervention effects, taking into account possible baseline differences, interaction effects of groups by time were calculated. For physician and patient ratings, two-way analysis of variance (ANOVA) was used to test for interaction effects of group allocation and measurement time. This procedure is recommended, when pre-existing groups are analyzed to detect changes [32]. Specifically, this procedure provides evidence for whether physicians or patients in the intervention group benefit over time compared to the control group. Because of the exploratory nature of our study, no multiplicity adjustment was applied. The level of significance was set to p < 0.05. To detect potentially relevant tendencies, we additionally considered the two-sided 10-percent level of significance (p < 0.1). This second significance level was chosen to conform with the argument that for the detection of intended changes in complex social systems lower aspirations should be acceptable and less vigorous standards should be targeted [17].

## Results

### Intervention

Physicians of the two IDs identified several work-related problems and aimed to implement respective solutions. During the 10-month duration of the intervention, in each ID, 11 meetings (à 1.5 hours duration) were held, in which three department physicians regularly participated. Common procedures of the meetings were: Problem analysis and identification of obstacles, identifying potential solutions, planning and scheduling the implementation, evaluation criteria, and timeline [22]. During following meetings, evaluation of progress and adjustments of solutions were undertaken. Additionally, a project steering committee (consisting of ID’s head physicians, the hospital’s managing director, and the medical director) met twice during the study period. Table 1 lists categorized problems and ten respective self-designed solutions.

**Table 1:** Hospital physicians’ work problems and self-developed solutions

**Problem category**	**Physicians’ solutions for implementation**
Work organization	- Joint ward rounds to improve mutual coordination of physicians’ and nurses’ work schedules during mornings,
	- Redirection of external telephone calls to reduce unnecessary workflow interruptions (to head physicians’ secretary),
	- Regularly scheduled consultation hours for relatives of patients in the afternoon,
Leadership quality	- Re-implementation of annual performance feedback and appraisal interview through departments’ head physicians,
	- Enhanced presence of supervisors on wards,
Internal information flow and quality	- Enhanced transparency of time documentation and subsequent salary accounting,
	- Implementation of department electronic whiteboard to spread information,
Qualification and training	- Inner-departmental schedule for specialty training,
	- Enhanced practice of case conferences in the department to discuss current patients,
	- Access online libraries; financial support of external training visits

### Qualitative data: implementation status within timeline and process-related factors

To assess the implementation status, semi-structured interviews with involved physicians and heads of the intervention department were conducted. Qualitative data indicated that the solutions proposed in the health circles (cf., Table 1) were implemented to different degrees within the two IDs. In Cardiology almost all solutions were regarded as implemented (exception: enhanced presence of supervisors on wards). In the Trauma department, a less vigorous implementation process co-occurred with more limited achievements, especially with regard to coordination of physicians’ and nurses’ daily work routines and the inner-departmental support for qualification and training. However, in both departments, improved leadership (e.g. re-implementation of annual performance feedback), enhanced technical support for information transfer, and increased qualification and training activities were reported by physicians. According to interviews, the following aspects of the intervention were perceived as favourable, advantageous, or beneficial: (1) Independent set up of the project agenda; (2) Support by a steering committee; (3) Continuous information flow; and (4) Back-up and support from the head physician within the department.

### Quantitative data: hospital physicians work organization and quality of care

In the following, the intervention effects with regard to work organization (primary outcome) and its impact on patient quality of care (secondary outcome) are reported.

### Subjects

At baseline, the sample size slightly differed from study onset, as 35 physicians in ID and 27 physicians in CD were eligible. The response rate was 59.5% (ID) and 70.8% (CD) respectively (Table 2). Similar numbers and participation rates were observed at follow-up. But only a minority of physicians was present at both waves of assessment, due to a high degree of professional mobility and turnover (ID: 42.9%, CD: 44.4%). In ID, N = 9 physicians reported at both time points, in CD N = 9 physicians were repeat responders.

**Table 2:** Study groups and response rate (for intervention and control departments across time)

		** Baseline (T1)**			** Follow-up (T2)**		
*Surveyed groups*		*Eligible physicians*	*Participating physicians*	*%*	*Physicians employed*	*Participating physicians*	*%*
**Physicians**	**Intervention**	35	19	54.3	37	21	56.8
	**Control**	27	19	70.0	25	16	64.0
		*Admitted patients*	*Participating patients*	*%*	*Admitted patients*	*Participating patients*	*%*
**Patients**	**Intervention**	1009	437	43.3	1027	621	60.5
	**Control**	751	305	40.6	805	296	36.8

Note: Intervention (Trauma Surgery and Cardiology Department), Control (General Surgery and Gastroenterology); Admitted patients: in patients who were admitted to the study departments within the timeline of respective assessments.

Patient participation rates are given in Table 2, with fair comparability between intervention and control departments, although patients in ID had a higher participation rate at follow-up.

To check for potential differences between study groups, baseline characteristics of participants were analysed. Overall, personal characteristics of physicians in both groups were comparable (cf., Table 3). A significant effect was only observed for gender.

**Table 3:** Demographic characteristics for physicians’ group at baseline and for patient groups at baseline and follow-up

			**Time**	**Intervention departments**	**Control departments**	**Test for difference**
**Physicians**	Age in years (M,SD)		T1	39.11; 7.6	40.65; 10.7	(t = -0.49; df = 33); n.s.
	Organizational tenure in years (M, SD)		T1	7.42; 4.68	8.14; 8.37	(t = -0.33; df = 35); n.s.
	Gender, male (N,%)		T1	16 (84.2)	10 (52.6)	**(Chi**^**2**^ = 4.39; df = 1); p = .04*
	Position (%)	(1)	T1	6 (31.6)	4 (23.5)	(Chi2 = 0.99; df = 1); n.s.
		(2)	T1	7 (36.8)	5 (29.4)	
		(3)	T1	6 (31.6)	8 (47.1)	
**Inpatients**	Age in years (M, SD)		T1	57.56; 17.23	63.71; 15.98	(F = 2.52; df = 697); n.s.
			T2	58.37; 17.82	64.24; 16.96	(F = 1.35; df = 890); n.s.
	Length of hospital stay in days (M, SD)		T1	7.44; 7.90	6.86; 5.92	(F = 0.06; df = 649); n.s.
			T2	6.48; 7.21	6.41; 5.79	(F = 0.05; df = 851); n.s.
	Gender, male (N,%)		T1	194 (46.85)	169 (58.48)	**(Chi**^**2**^ **= 8.73; df = 1); p < .01**
			T2	282 (46.53)	137 (48.92)	(Chi^2^ = 0.06; df = 1); n.s.

Note: T1 baseline, T2 follow-up; Physicians T1: N = 38; Patients T1 N = 742 and T2 N = 917; M Mean, SD Standard Deviation, p Significance level; Coding of Position: (1) Department Head & Senior Physicians, (2) Specialists, (3) Assistant Physician (entry level).

Regarding patient characteristics, self-reported mean age and length of stay in the hospital did not differ between the two measurement points (cf., Table 3). Gender only differed significantly between ID and CD at T1. No such difference was observed at follow-up.

### Outcome I: hospital physicians work organization

Hospital physicians reported working conditions are displayed in Table 4. We tested first for baseline differences between the intervention and control groups. For the six indicators, only one significant mean difference was found: Colleague Support was higher in CD than in ID: t(df = 36) = -2.00, p = .05. Next, we analysed within-group changes over time. Within the intervention group, substantial improvements were observed, but differences were significant only in case of “conflicting demands”: t(df = 38) = 2.93, p = .01. Within CD, no similar changes were observed. Next, we tested for differences between intervention and control group at follow-up. Significantly less conflicting demands were reported within the ID compared to the CD: t(df = 36) = -2.00, p = .05.

**Table 4:** Physicians’ work organization for group and over time

		**Intervention departments**				**Control departments**				**Significance testing**
		**Baseline**		**Follow-up**		**Baseline**		**Follow-up**		**Group x Time**
		**N = 19**		**N = 21**		**N = 19**		**N = 16**		**(ANOVA)**
**Indicators of hospital physicians’ work conditions**		**M**	**SD**	**M**	**SD**	**M**	**SD**	**M**	**SD**	**F(df = 1); p**
1	Workflow interruptions	4.04	.67	3.77	.68	4.05	.59	4.06	.47	0.69; n. s.
2	Conflicts in role and task demands	3.42	.60	2.83	.67	3.30	.82	3.27	.64	**3.03; .09**^**†**^
3	Colleague support	3.11	.77	3.38	.74	3.58	.68	3.31	.46	**2.91; .09**^**†**^
4	Quality losses	3.12	.95	2.83	.73	2.98	.93	2.83	.74	0.54; n. s.
5	Quality of cooperation with patient relatives	3.47	.51	3.71	.56	3.74	.65	3.44	.63	3.90; .05*
6	Quality of cooperation with nursing staff	3.74	.73	4.05	.59	4.00	.88	3.81	.66	2.21; n. s.

Note: N Number of Physicians, M Mean, SD Standard deviation, Scale Range: Scale of indicators 1-4 1 = “not at all” to 5 = “yes, to a very great extent”; Scale of Indicators 5 and 6 1 = “very bad” to 5 = “very good”; *p < .05 Significance level, †p < .10 Significance level.

Furthermore, to test for interaction effects of group allocation and measurement time, we conducted two-way analyses of variance with interaction terms (cf., Table 4). For one of the six indicators, “change in quality of cooperation with patients’ relatives”, we found a significant improvement in the ID; additionally, in two further indicators, “conflicting demands” and “colleague support”, tendencies in the expected direction were observed.

Since a minority of physicians (ID N = 9, CD N = 9) participated in both measurement waves, we conducted additional analyses to investigate their evaluations over time. Results are presented in Additional file 1: Table S1 and mainly support the above reported findings, also with regard to conventional classification for effect sizes; d = .2 to .4: small, d = .5 to .7 medium, and > .8 large effect [33]. In the ID, a large effect could be observed for decreased conflicting demands and medium effects for increased colleague support, improved cooperation with relatives, and decreased quality losses. In the CDs, only one substantial effect could be observed, such that workflow interruptions decreased over time (cf. to Additional file 1: Table S1).

### Outcome II: patient care quality

The effect of the intervention on the quality of patient care was analysed based on ratings in the patient survey (cf., Table 5). We first tested for between-group differences at baseline: For “Organization of Physicians’ Care” no differences were observed, but “Quality of Information from Physicians” was rated more favourably in the ID: t(df = 690) = 2.90, p < .01. Next, we checked for within-group changes over time. In the ID, “Quality of Information from physicians” improved over time with marginally significant level: t(df = 1017) = -1.63, p = .10. In the CD, “Physicians’ Organization” tended to be rated less favourably. Thus, some between-group differences are significant at the post-intervention assessment: Patients in the ID rated “Organization of Physicians’ Care” more positive than patients in the CD: t(df = 883) = 3.99, p < .01. Similarly, “Quality of Information from physicians” was judged to be significantly higher for the ID: t(df = 876) = 3.92, p < .01. Finally, testing for interaction effects of group allocation over time, an interesting tendency was observed (cf., Table 5). Specifically, results suggest that the overall change of perceived quality of physicians organization of care can be attributed to the allocation of the improvement intervention. The results also indicate that perceived quality of care decreased in the CD (cf., Table 5).

**Table 5:** Patients’ rating of care quality over groups and study time

	**Intervention departments**				**Control departments**				**Significance tests**
	**Baseline**		**Follow-up**		**Baseline**		**Follow-up**		**Group x Time**
	**N = 410**		**N = 604**		**N = 286**		**N = 281**		**(ANOVA)**
**Indicators of patient care quality**	**M**	**SD**	**M**	**SD**	**M**	**SD**	**M**	**SD**	**F(df = 1); p**
1 Organization of physicians’ care	4.20	.82	4.23	.75	4.13	.76	4.01	.84	**3.67; .056†**
2 Quality of information from physicians	4.08	.83	4.16	.77	3.88	.88	3.93	.81	0.14; n. s.

Note: *N*, Number of patients; *M*, Mean; *SD*, Standard deviation; Scale Range 1 = “not at all” 5 = “yes, very much”; †p < .10 Significance level.

## Discussion

In this controlled, randomized intervention study, we analysed whether physicians working conditions in surgical and medical departments of a hospital can be improved through a participatory work re-design intervention. We additionally explored related improvements of physician performance, as reflected by patient judgments regarding the quality of patient care. The study shows that for several prevalent problems in the physician’s hospital working environment, solutions can be found and successfully implemented. Overall, the study results indicate positive effects of the intervention on conflicts in role and tasks demands, colleague support, and quality of cooperation with patients relatives. At the end of the intervention, patients of the ID rated the organization of patient care more favourably than patients in the CD.

An intervention to change hospital physician working conditions is subject to complex influences, which are only partly under the control of the researchers [34]. Additional information is needed to shed more light on process-related factors [16,17,20]. Therefore, supplementary interviews with involved physicians served to identify important procedural aspects to enhance opportunities for transferring results into practice and guide further research [16,20,35]. The interviews revealed that the hospital physicians rated the intervention process positively.

Although differences between ID and CD regarding study outcomes mostly attained only marginal significance (p < 0.1) and effect sizes were rather small, obtained results are plausible with regard to the implemented changes in work design and consistent with one another. The fact that improvements were observed from two independent perspectives (physicians and patients) increases the validity of our results and, thus, supports the usefulness of similar interventions in organizational development projects. In line with previous research findings, it appears overly optimistic to expect consistent and enduring positive effects of a single organizational intervention, which was limited in scope [16,20]. However, even small changes of specific stressful working conditions (such as reduced interruptions and conflicting demands, improved coordination, and support among teams) may be beneficial in the long run.

### Study’s strengths and limitations

To our best knowledge, this is the first controlled intervention study with the aim of improving working conditions and quality of care of hospital physicians by applying a participatory change process. Conducting such a study proves to be highly challenging, given the complexities and dynamics of hospital settings. It is therefore no surprise that only few studies have addressed this topic so far [11]. By applying cluster randomization, measuring the core variables with standardized, validated scales, and testing intervention effects with established statistical methods, we were able to demonstrate the feasibility, scientific relevance, and potential practical usefulness of this approach [29,36]. A second strength concerns the inclusion of a large number of patient evaluations of physician performance. This is rarely done, despite the obvious importance of the consumer perspective in health care. The combination of data from two stakeholder groups strengthens the validity and robustness of our findings. Third, our additional qualitative information enriches outcome data with process-related details and thus contributes to an in-depth interpretation of observed changes in attitudes and behaviours [17,37].

There are several limitations to this study. Firstly, the restriction to one single hospital setting limits the generalization of the results as well as the applicability of the implemented organizational changes and effects beyond this hospital. There is, however, reason to assume that the identified core dynamics are similar in other hospitals of this type, given the commonalities of tasks and similar overarching organizational constraints. Furthermore, an additional investigation of a larger sample of German hospital physicians revealed a high degree of concordance with our physician-based findings [38]. Secondly, the number of physicians included was relatively small, thus compromising the statistical power. It was not possible to reduce the regular turnover of physicians during the intervention, given the constraints of their training careers and rotating schedules. In ID, 2 to 3 physicians regularly took part in actual intervention meetings, and subsequently discussed or informed their colleagues about problems, solutions and implementation status (usually during routine departmental meetings). Thus, we cannot estimate to what extent the individual physicians were exposed to the intervention (i.e., dose delivered). This is particularly relevant since physicians who may have felt little involvement in the intervention initiatives may have reported unchanged working conditions [16].

Although physicians were not explicitly informed about study hypotheses in detail, the broader objective to improve working conditions was communicated openly. Since it is not feasible to blind employees in a participatory intervention, bias may occur if participants respond differently with regard to study objectives. For example, positive developments may have been more salient in the ID than in the CD. Conversely, announcing the intention to improve working conditions may also have led to expectancy effects, resulting in a more critical response tendency in the ID. Further, in the CD, both lower response tendencies due to the relative deprivation compared to the ID as well as more optimistic ratings based on psychological compensation processes are theoretically plausible. Overall, while we cannot rule out effects of the unavoidable lack of blinding, there is no clear indication that these would necessarily inflate the observed differences in change between study groups.

Furthermore, the observed positive intervention pattern in patients’ reports supports our findings, because patients were completely blind to the design of the intervention and as such were not able to respond according to the study objectives.

In our statistical analysis of intervention effects, we treated time and group affiliation as independent factors, since the number of physicians that participated in both waves was low (N = 9, respectively). To account for potential bias due to repeated measurement, future intervention studies should seek to recruit larger samples to allow robust statistical analyses, e.g. mixed-model design ANOVA. To address potential bias due to the repeated participation of physicians at baseline and follow-up, we conducted an in-depth analysis for this particular subgroup (cf. Additional file 1: Table S1). Furthermore, while differing medical specialties make ‘contamination’ (i.e., personnel changes) between the ID and CD unlikely, we cannot definitely exclude such an effect.

A third limitation concerns the scope of the intervention applied. We restricted the measures to distinct organizational changes at the level of single departments. This restriction precludes changes depending on higher-level decision making. In highly complex, multifaceted organizations such as hospitals, some problems, e.g. concerning personnel resources allocation or other broader organizational practices, need to be addressed at higher organizational levels. Although the inclusion of the steering committee aimed to address this issue, the effectiveness of some changes implemented at the departmental level was weakened due to this limitation (e.g., lack of momentum). Likewise, while focusing on structural measures, our approach did not target individual-level behavioural changes. It is possible that by combining organization-based interventions with person-based interventions, stronger effects may have been observed [17,18,39]. Yet, it is also possible that our intervention strategy may produce more robust effects in the longer run, and that the observation period of potential changes was too short [16,40].

Two less tangible limitations relate to unmeasured effects evolving from cluster-randomization and to potential bias in the patient survey. Randomizing departments instead of individuals introduces the risk of neglecting variations in organizational culture and leadership style which may influence the implementation. Concerning the patient samples, we observed similar participation rates at study entry, but a considerably lower participation rate in the CD at follow-up. Due to confidentiality regulations we were not able to check for detailed patient characteristics that may shed light on a potential response bias [41]. Overall, no clear biasing tendency can be derived from the reduced survey participation of patients in the CD at follow-up, which could be based on selection effects related to either low or high satisfaction with the delivered quality of care. Additionally, while we cannot exclude that few patients were included in both measurement waves; this poses a limited risk for bias due to the rather large sample sizes.

## Conclusions

Hospital physicians work life and well-being are critical assets in ensuring a well-functioning health care system [1]. Our study provides preliminary evidence of the effectiveness of a participatory change process aimed at improving physicians working conditions and quality of patient care. Although effects were rather modest, pointing towards a need for further larger trials, our findings are useful in informing and guiding structural improvements of hospital physicians work life.

### Ethical approval

The study was approved by the Ethics Committee of the Faculty of Medicine of Munich University (No. 124-07).

## Competing interests

The authors declare that they have no competing interests.

## Authors’ contributions

All authors jointly designed the hypothesis, analysed and interpreted the data, and wrote the manuscript. All authors read and approved the final manuscript.

## Pre-publication history

The pre-publication history for this paper can be accessed here:

http://www.biomedcentral.com/1472-6963/13/401/prepub

## Supplementary Material

Additional file 1: Table S1 Physicians’ work organization for N = 18 physicians who participated at baseline and follow-up. Click here for file
