# Supplementary material for: The effects of improving hospital physicians working conditions on patient care: a prospective, controlled intervention study
Source: BMC Health Serv Res. 2013 Oct 9;13:401. doi: 10.1186/1472-6963-13-401 (PMC3851860; doi:10.1186/1472-6963-13-401)
Supplement: Additional file 1: Table S1 — Physicians’ work organization for N = 18 physicians who participated at baseline and follow-up. [file 1472-6963-13-401-S1.doc]

# Additional Files

### Table S1: Physicians’ work organization for N=18 physicians who participated at baseline and follow-up

|  | | **Intervention Departments (N=9)** | | | | | | |  | **Control Departments (N=9)** | | | | | | |
| --- | --- | --- | --- | --- | --- | --- | --- | --- | --- | --- | --- | --- | --- | --- | --- | --- |
|  | | Baseline | |  | Follow-up | |  |  |  | Baseline | |  | Follow-up | |  | |
| Indicators of hospital physicians’ work conditions | | M | SD |  | M | SD | Wilcoxon test for  mean difference (p) | Effect size (d) |  | M | SD |  | M | SD | Wilcoxon test for  mean difference (p) | Effect size (d) |
| 1 | Workflow interruptions | 4.04 | .63 |  | 3.92 | .73 | n. s. | .18 |  | 4.48 | .41 |  | 4.15 | .53 | n. s. | .69 |
| 2 | Conflicts in role and task demands | 3.28 | .66 |  | 2.69 | .74 | **.09†** | .84 |  | 3.36 | .78 |  | 3.41 | .95 | n. s. | .06 |
| 3 | Colleague support | 3.15 | .75 |  | 3.59 | .81 | **.03** | .56 |  | 3.30 | .68 |  | 3.48 | .50 | n. s. | .30 |
| 4 | Quality losses | 3.15 | .97 |  | 2.63 | .82 | n. s. | .58 |  | 2.86 | 1.09 |  | 2.78 | .90 | n. s. | .08 |
| 5 | Quality of cooperation with patient relatives | 3.44 | .53 |  | 3.67 | .50 | n. s. | .45 |  | 3.44 | .73 |  | 3.56 | .53 | n. s. | .19 |
| 6 | Quality of cooperation with nursing staff | 3.89 | .93 |  | 4.11 | .60 | n. s. | .28 |  | 4.00 | .87 |  | 3.78 | .83 | n. s. | .26 |

Note: N Number of Physicians, M Mean, SD Standard deviation, Scale Range: Scale of indicators 1-4 1=”not at all” to 5=”yes, to a very great extent”; Scale of Indicators 5 and 6 1=”very bad” to 5=”very good”; bold p<.05, †p <.10 Significance level; Cohen’s d: effect size.
